# Tanzanian Nurses Understanding and Practice of Spiritual Care

**DOI:** 10.5402/2011/534803

**Published:** 2011-06-06

**Authors:** Khairunnisa Aziz Dhamani, Pauline Paul, Joanne Kaye Olson

**Affiliations:** ^1^Tanzania Institute of Higher Education, The Aga Khan University, P.O. Box 38129, Dar es Salaam, Tanzania; ^2^Faculty of Nursing, University of Alberta, 3rd Floor Clinical Sciences Building, Edmonton, AB, Canada T6G 2G3

## Abstract

Spirituality, as a basic characteristic of humans and a contributor to human health, is regarded as part of nursing practice. The purpose of this study was to examine how Tanzanian nurses understand spirituality and spiritual care. Using the qualitative method of interpretive description, fifteen registered nurses engaged in clinical practice in a Tanzanian hospital were recruited to participate in this study. In-depth interviews using open-ended questions were carried out, tape-recorded, and transcribed verbatim. Data collection and inductive analysis occurred concurrently. In this paper, key findings are grouped under the following headings: meaning of spiritual care, recognition of spiritual needs, and interventions to respond to spiritual needs. Although there were some differences, overall participants' understanding of spirituality and spiritual care was similar to what is found in the literature about nurses in other countries. The provision of spiritual care also included some unique elements that may reflect the African context.

## 1. Tanzanian Nurses Understanding and Practice of Spiritual Care

There is growing evidence in the literature to suggest that attention to spirituality positively influences the ability to cope with illness, the prevention of illness [1], the ability to find meaning and purpose in life, and overall well-being [2–4]. Life-threatening situations sometimes give rise to complex spiritual questions [1, 2, 5], and patients may wish to address these questions with nurses. Therefore, it is important for nurses to be knowledgeable about and prepared to deal with the spiritual component of care so they can support clients in times of need. 

Spirituality is an integral part of a person's wholeness [6–8]. According to the holistic paradigm, body, mind, and spirit are considered intertwined [9], and every human experience, including health and episodes of illness, can be considered spiritual experiences. Similarly, in the African context, spirituality is considered an integral part of the person and traditional health practices tend to be holistic [10, 11]. 

Taylor [8] has defined spiritual care as an approach used to integrate all aspects of a human being. Spiritual care is similar to holistic care where the physical, psychological, social, and spiritual dimensions of an individual are considered as a whole [12]. Professional nursing practice standards used in Tanzania, Canada, and the United States include statements about the responsibility of registered nurses to provide spiritual care [13–15]. 

Spirituality and spiritual care in nursing practice have primarily been examined by researchers who live in western countries and with research participants who are also from these countries. The purpose of this study, the first in the context of Tanzania, was to explore how nurses from this country understand spirituality and the provision of spiritual care. The research questions were the following (1) How do Tanzanian nurses describe/understand the concepts of spirituality and spiritual care? (2) How do Tanzanian nurses practice spiritual care? In this paper, we primarily focus on the second question. 

## 2. Methods

This study followed the interpretive descriptive approach, a method designed to answer specific questions related to practical aspects of nursing [16–18]. This method includes characteristics that are informed by axioms from  Lincoln and Guba's naturalistic inquiry tradition [16, 18, 19]. The key axioms are as follows: (a) multiple realities are constructed in the context of complex human experience and may well be contradictory; thus, reality is subjective; (b) because the researcher (inquirer) and the participant (object of inquiry) interact and influence one another, they are inseparable; (c) theory must emerge from the data rather than using an a priori theory; (d) the focus is on human commonalities and individual variance within the area of inquiry; (e) inquiries are conducted in as naturalistic a context as possible to maintain the respect, comfort, and ethical rights of all participants [16, 18, 19]. An interpretive description produces constructed truths related to spirituality and spiritual care rather than facts because of the reliance on interpretation. The context in which the knowledge related to spirituality and spiritual care was generated in this study was Tanzanian nurses' perspectives. Ethical approval was obtained in Canada and Tanzania prior to data collection. 

### 2.1. Sampling and Setting

Fifteen study participants were recruited from  one Tanzanian hospital to provide  meaningful descriptions of the concepts of *spirituality* and *spiritual care*. Advertisement flyers, which included information about the study and how to contact the first author, were posted on the notice boards of the wards of the hospital. During the data collection period, there was no situation where we had to refuse participants in enroll in the study. 

We purposively drew the sample to capture expected and emerging variations within the study concepts [17, 18, 20]. Purposive sampling involved selecting registered nurses who were directly engaged in clinical practice, had more than one year of clinical experience, and were willing to articulate their thoughts about spirituality and spiritual care. To achieve maximum variation in sampling, we ensured that participants varied in gender, religious affiliation, years of clinical experience, professional qualifications, and area of work. All participants were over 26 years; 73% were females, 27% were male, 80% were Christians, and 20% were Muslims. The participants were not asked about their religion or the denomination of a religion, but they self-identified their religious affiliation. In Tanzania, Muslims and Christians are approximately equal in number but there are no available statistics on the religious affiliation of nurses. Fifty three percent of study participants held a BScN, and 47% were diploma prepared. Participants' clinical experience ranged from 1 to 38 years and represented a variety of nursing units such as maternity, medical, surgical, operating room, critical care, paediatrics, and emergency. 

### 2.2. Data Collection

The primary data-collection strategy was in-depth face-to-face semistructured interviews. An interview guide consisting of semistructured and open-ended questions was developed to elicit a description of spirituality, spiritual care, and spiritual caregiving practices. In addition, the questions also addressed the hindering and enabling factors for the integration of spirituality into nursing practice and traditional healing practices. All the interviews were audio-taped on a digital ICD recorder with the participants' permission and were transcribed verbatim. Interviews ranged from 45 to 90 minutes. Field notes were maintained immediately after each interview. Interviews and transcriptions were conducted by the first author, while analysis was carried out by all three authors. 

### 2.3. Data Analysis and Rigor

Data collection and analysis occurred concurrently. The critical review of the literature/knowledge related to nurses' spirituality and spiritual care formed the basis for the preliminary analytic framework. Interpretive description gives to the researcher the freedom to utilize coding approaches from other forms of qualitative research methods. Coding was guided by Creswell's [21, pages 191–195] generic steps of data analysis. As recommended by Creswell [21], initial analysis took place by reading and rereading the transcripts and gaining an understanding of the overall picture of the phenomena under study. Detailed analysis began with a coding process. The coding process involved organizing participants' sentences and paragraphs with similar properties into categories. Several categories evolved from the analysis of data. Broad-based in vivo codes were assigned to each of the categories generated. Later, data content belonging to each category was grouped in one place before performing preliminary analysis. 

Categories that characterized spirituality, spiritual care, and other related phenomena were grouped together into themes. These themes represented the major findings of this qualitative study. The initial themes were tested and refined in subsequent interviews with research participants throughout the study.

Journal writing, field notes, and prolonged immersion with the data, along with researchers knowledgeable in the area of spirituality and data analysis, enhanced *representative credibility* of the study [22–24]. The credibility criterion of *epistemological integrity* was established by selecting data sources and interpretive strategies based on the research questions and the level of existing knowledge about the phenomena under study, and *analytic logic* by maintaining an audit trail. The criterion of *interpretive authority* was established by describing participants' narratives in detail before labelling categories and themes and by having extensive discussion about the findings and data analysis. 

## 3. Results

The results presented here focus on the meaning of spiritual care, recognition of spiritual needs, and interventions to respond to spiritual needs. To protect the confidentiality of the participants, we have used pseudonyms to identify their quotes. 

### 3.1. Meaning of Spiritual Care

For a majority of the participants, spiritual care was defined within the contexts of religious practice, holistic care, and healing practices. For the participants, spiritual care meant incorporating religious practices or beliefs into the provision of nursing care. As stated by Angelina, “spiritual care means that somebody is incorporating the belief of spirituality into their care. Spiritual care is adhering to your client's beliefs about God.” Likewise, Natalia expressed spiritual care as “incorporating religious beliefs when reassuring and counselling the patient.” Esther described spiritual care as

the part of the care, which touches the inner part of the person, the unseen area of this person and the faith of the person (pause) which touches that area which will give worth to people, give this person a positive outlook on life even if this person failed to be cured but there is always hope and positive expectations.

There were some nurses who believed that spiritual care encompassed physical and psychological care in addition to integrating religious practices. “Spiritual care is body care and psychological care” including encouraging clients to trust God (Michael). Likewise, Ombeni mentioned, “Our day to day activities are spiritual care in addition to providing religious care.” Participants viewed holistic care as recognition of all dimensions of an individual—physical, psychological, social, and spiritual. Isaac said

There is a connection between physical, psychological, and spiritual aspects. There is a deep connection. Though spiritual care and the rest of the care are a bit apart in understanding, they need to be understood to be one thing, they should come close, (they) need to be one thing.

Karen eloquently addressed the holistic nature of nursing by stating

Spiritual, physical, psychological they all can be brought in together or merged into one basket when caring for a client in need and to be used right;… I mean, in day-to-day working, you really need to involve spiritual care so that the whole basket can be full.

The participants in this study were less vocal about the social dimension when describing the holistic nature of nursing.  However, they realized that attending to social needs is an important aspect of holistic care. For example, Bora said

Sometimes we get a patient with a social problem, so I stay there, talk with her and listen to the problem. If she does not remember God, if she does not know the words from the Bible and I know that there is a verse that will help her psychologically, I ask her to read those lines from the verses and sometimes she feels better. To help with social problems or psychological problems we need the spiritual support.

Within the holistic nature of nursing care, the participants alluded to the connection between healing and spirituality. They believed that healing was a spiritual process and the focus of nursing care. Esther said

Nursing is more about healing rather than curing. I am saying this because you might have an illness and go to a doctor, and you expect to be cured, but the healing part is more inside, something which is within someone, so something which you cannot get to through medicines only, or through what nurses are doing. It is something inward. So if someone is healing it is the spiritual area that is touched. Sometimes people lose hope. When you touch the spirit part of this person, this person feels, “now at least I have someone else, (I'm) not alone.” When someone has this will-power and the spirit, it assists a lot in curing the body. This is the area which I think is very important in nursing, where spirit is concerned.

However, Humphrey believed that nurses usually attend to curing the body while healing is performed by clergy. He said

A patient is admitted to the hospital, he or she is sick and when you give the treatment it means that we want to cure, it is like curing. Healing is related to spirituality, I mean it occurs through these church people (pause); we say that God heals people. Mostly we nurses focus on the curing part.

### 3.2. Recognition of Spiritual Needs

Communication, the health status of patients, beliefs in witchcraft and devils, expressions of feelings, and close observation of the environment were identified as means of recognizing spiritual needs of patients. Bora said, “It is good to know patient's religion and patient's views about religion. It is important to know how frequently patients attend church, so it is good to know that background before you (nurse) can use spiritual care interventions.” Similarly, Angelina mentioned that, “maybe through (the health) history you know if he believes in any God, or what.” Karen said her clients would point out spiritual needs by requesting “Can I have  my religious leader come over as I need to share something?” For Fiona, when her patient would request a pastor, to do confession, it indicated a spiritual need of the patient. Participants stated that when their patients were found terminally ill, dying, or hospitalized for a long period of time it often indicated a spiritual need. Esther said

It is like a dying patient because we are nurses we know that, of course, we are not God, but there are some illnesses where you find that nothing can be done (pause), this person's end is coming. You find that this patient is struggling for life, this patient is screaming from pain so I need to sit and talk to this patient. So you find that need.

Some participants considered patients newly diagnosed with HIV/AIDS and patients undergoing surgeries such as amputation or removal of some other body parts as in need of spiritual care. Natalia said “she was tested for HIV and she was positive and she was very sad because she did not think that she would have it…. What we did was to call the pastor after talking with her.” Laurencia indicated that most of the women in labour would request nurses to pray for easy delivery and normal birth of a child.  Most of the participants agreed that every patient required spiritual care but due to lack of time and staff, it was difficult to reach out to everyone. The other indication that made nurses aware of spiritual needs was when a patient expressed beliefs in witchcraft or devils. For example, Fiona stated

Once the patient is there, you care for him, you go to the bed, you talk with the patient but the patient does not respond to anything, or she just responds slowly…. Some of the patients are shouting, “I am dying, dying, there is somebody who is following me,” (nurse says) “what?” So we know that there is a spiritual need. They say that, “I am getting treatment, I am not going to be cured, I am bewitched.”

Similarly Jumma stated “Traditional healers say that they are talking to devils and the devil responds saying that the patient has this problem… So when the relatives do what has been said by the devil, the patient may be cured.” Many participants in this study believed that the diligent observation of their patient's environment and the expression of feelings by their patients significantly made them aware of the patients' spiritual needs. Karen said

This patient was alone in that room. I went in and I found her crying… I took her hand and asked, “What is wrong? Why are you crying?” She went on sobbing…. So those are the moments where we have planned for that spiritual care.

Ghanima described a patient's bedside environment which was indicative of spiritual needs. She stated that

She (patient) was very old, she had had a CVA (cerebrovascular accident) for a long time, almost a year. She had been hospitalized many times…. We cared for her in the ward and always when you went in her room, you would find a candle, you would find a picture of Jesus around her bed. Whenever we did bed making or whatever, we placed everything as the relatives wanted it to be. They said that she felt better when we put things around her.

### 3.3. Interventions to Respond to Spiritual Needs

The nurse participants in this study described religious- and non-religious-based spiritual interventions. Following are some of the interventions described by participants.

#### 3.3.1. Religion-Based Spiritual Care Interventions

**Facilitating and Conducting Prayers**

All participants in this study strongly expressed that prayers were the most common way of providing spiritual care. Celeste believed that “Prayers help in strengthening one's faith and spirituality; they help in enhancing hope and bringing harmony among mind, soul, and body, and consequently in attaining serenity and inner healing.” Karen expressed the importance of prayers for her ICU patients, “While you are giving care to that particular patient, why not speak to that person about God? Why not say that God cares about his or her life and that He will be there to rescue him or her.” Ghanima facilitated prayers for patients by finding space and time for praying. If patients' religious affiliations were different from those of the participants, they relied on colleagues with backgrounds similar to the patients. However, some participants thought that it was a challenge to incorporate spiritual care if patients belonged to a different faith. In the case of Humphrey, he denied giving spiritual care to his patients based on his understanding that he did not incorporate prayers in his care.

**Reading of Holy Scriptures**

Some participants thought that reading Holy Scriptures promoted hope and peace amongst their patients. Bora illustrated the importance of reading the Bible:

If we believe in those words  from the Bible, automatically it can change somebody spiritually. I have to communicate with her or him and try to speak to them about the words from the Bible, and to reassure her or him according to his or her problem. Usually I walk with my Bible in the ward and patients can use the copy of my Bible. I tell them, “read here this line it will help you” and I think that is spiritual care.

Laurencia mentioned that when she finds time, she sits with her Christian patients, reads, and refers to relevant passages from the Bible. Participants also encouraged Muslim patients to read the Quran but bringing them a copy was difficult because there were no copies of the Quran available in the hospital.

**Pastor/Imam Consultation**

Organizing a visit from a pastor (or priest) or an imam was considered a spiritual care intervention for the majority of the participants. Angelina said “I can ask for permission from a patient and consult the priest. I have seen many patients bringing a priest to the hospital to pray for them”. Esther said “Priests talk to the patients… if they wish he can pray for them and if someone says “no,” then they do not insist. If someone says “yes,” whether a Muslim or a Christian they pray.” Ghanima mentioned that an imam can be included in patient care.

**Encouraging Patients to Trust God/Supreme Being/Creator**

Bora said, “Believe that there is a God, and God will solve this problem because it is now out of your capacity, explain your problem to God and He will solve it.” Angelina mentioned, “When providing spiritual care, maybe I say to a patient, “do not worry, God will take care of all these things, just believe that God will take care of people at home and God will take care of you here.”

#### 3.3.2. Non-Religious-Based Spiritual Care Interventions

**Demonstration of Love, Compassion, and Forgiveness**

For example, Dorothea said

Patients represent God, so when attending anybody it means you are attending to God, because we are not seeing God, the one you are seeing is representing God, so you must show him love, he needs care and you need to be patient towards him.

In relation to forgiveness, Celeste stated “Forgiveness gives inner peace and calmness  ... it sets people free spiritually for both the forgiver and the one being forgiven.” Esther stated that taking interest in patients was also part of spiritual care interventions. Isaac said that listening to a patient actively and being present were important aspects of a human interaction. He said “One must listen to a patient, always remain present when caring for a person. Also, one needs to communicate very well when a patient comes with some fears and be present during the time of sorrow.” Michael believed that demonstrating empathy and sympathy helps patients cope with illness.

**Maintaining and Demonstrating Moral and Ethical Behaviour**

Some of the moral and ethical behaviours such as being polite, honest, faithful, and respectful of individuals regardless of religious beliefs were considered spiritual care interventions. Celeste said

The first step that I use is just showing that person that I care for you, I respect you as a human being, you are a unique person and I care for your needs, and I am open to listen to you and to help you as much as I can.

For Ombeni, human interaction in terms of honesty and faithfulness to patients was counted as spiritual care. She said

Because when we are doing our daily activities, we are supposed to be very faithful to our patients. Being faithful is spiritual because when the doctor writes the order let's say injection Rochephen and you are giving water for injection (distilled water used for dissolving parenteral medications)…. So being honest with patients is considered spiritual. Also, putting a patient in a conducive environment, making sure that the technology is working properly in ICU (intensive care unit), and there are enough lights. This is considered as providing spiritual care. In addition to providing religious care, our day to day activities are spiritual care.

**Counseling and Reassurance**

Many participants considered counselling and providing reassurance to patients as significant spiritual care interventions. Michael mentioned that “providing reassurance to patients, making a patient pain free, and reducing patients' fears related to surgery are part of spiritual care.” Natalia also mentioned that reassurance and counselling were part of spiritual care. She said

Spiritual care is reassuring and counselling the patient. If you do not do counselling you will not know that this patient has a spiritual problem. Also providing reassurance allays anxiety. Sometimes when you do counselling the patient may solve the problem.

## 4. Discussion

Participants' definitions of spiritual care are congruent with definitions presented in the literature that highlight nurses' perception of spiritual care as supporting religious beliefs and practices [25–27]. From participants' accounts, it can be implied that spiritual care was mainly understood from a religious framework. However, some participants provided evidence that their conceptualization of spiritual care interventions was broader and included being compassionate with patients, respecting them, fulfilling their needs, and listening to them. These findings support what has been described in the literature [28–32]. 

From the examples shared by participants, the identification of spiritual needs often included a process in which both patients and families expressed directly or indirectly a need for spiritual care. Participants shared that they identified spiritual needs by listening, talking, and observing the environment. The strategies were similar to those found in the literature [9, 28, 33]. 

More unique to the African context, the participants indicated that mentions of witchcraft and beliefs in devils were clues indicative of spiritual needs. This aspect of spiritual assessment is rarely discussed in the nursing literature. However, Murray and Zentner [34] include this topic as one to consider in assessing for spiritual needs. Literature describing traditional contexts provides information about the role of traditional healers in dealing with beliefs related to evil spirits [35–37].

In this study, spiritual interventions identified by participants included religious-based and non-religious-based interventions. Religious-based interventions included praying, reading Holy Scriptures, arranging for a visit with a pastor/imam, and supporting expressions of faith. Nonreligious interventions included demonstrating ethical behaviour, being compassionate, facilitating forgiveness, and providing counselling and reassurance. Findings of this study are generally consistent with the literature on spiritual caregiving practices that include a religious component [9, 12, 28, 34, 38–40]. Praying is considered useful in facilitating the process of healing and restoring hope in the midst of crisis [41]. According to Salman and Zoucha [42], prayers bring people closer to the divine, which in turn reduces the risk of depression, anxiety, and helplessness. However, Carson and Koenig [43, Page 146] warn that prayer should not be used as a substitute for health care provider's time or for meeting healthcare provider's needs or “to communicate a magical view of God that conveys a false sense of hope and expectation”. 

Another common spiritual care intervention expressed by participants was providing and/or reading Holy Scriptures. Numerous authors support the view that scripture reading may be a source of spiritual comfort for patients [34, 42, 44, 45]. Accepting someone without prejudice and judgment and respecting their expressions of faith allow for optimum interpersonal connection which may promote the healing process [46, 47]. Respecting and addressing client's spirituality and religious practices are expected of nurses [13, 15, 48, 49]. However, the findings in this study indicate that some of the participants may have considered that telling a patient to trust God is addressing spiritual needs, while in fact it may be imposing their own beliefs. 

Developing an understanding of the role and functions of pastors, imams, and chaplains assists nurses to better collaborate with these team members for improved spiritual care outcomes [48]. Nurses in this study primarily sought clergy to conduct prayers and perform confession. At the hospital where the study was conducted there were no chaplains, thus pastors and imams came from the community to offer their services. The absence of chaplains may in part explain nurse participants' focus on the religious aspects of spiritual care. Because chaplains have the preparation to specifically work in a health care environment within the health team [50], they can role model the provision of broad spiritual care. 

The study participants stated that demonstrating ethical behaviour and compassion towards patients was part of spiritual care interventions. According to Galek et al. [46] and Newshan [51], love, belonging, and respect create a sense of connectedness with others, and these attributes of spirituality contribute to patients' sense of comfort. Similarly, Emblen and Halstead [29] and Puchalski [52] propose that listening, being compassionate, and being present are attributes nurses require to provide spiritual care. 

Facilitating forgiveness was mentioned by the participants of this study. It is also recognized as a spiritual intervention by an increasing number of authors [53]. The fact that it is now included in the Nursing Intervention Classification (NIC) indicates acceptance as a nursing intervention [54]. Counselling and reassurance were other spiritual care interventions identified by participants. Murray and Zentner [34] also considered counselling to be part of spiritual care and suggested that the use of counselling can lead to healthier outcomes. 

## 5. Conclusion

The results of this study shed light on how Tanzanian nurses understand and practice spiritual care. Tanzanian nurses who participated in this study showed the desire to provide spiritual care, and much of their understanding was similar to the understanding of nurses in other parts of the world. It seems that these participants were engaging in religious-based spiritual care interventions more often than would have been the case in many other parts of the world. Findings of this study indicate that although there is a shared understanding of spirituality and spiritual care, contextual factors play a role in the type of interventions selected. This study is a first step in trying to understand how African nurses describe spirituality and spiritual interventions. Further research has the potential to expand knowledge in this area and will give direction for possible interventions that will affect nursing practice. 
